# Giving Leads to Happiness in Young Children

**DOI:** 10.1371/journal.pone.0039211

**Published:** 2012-06-14

**Authors:** Lara B. Aknin, J. Kiley Hamlin, Elizabeth W. Dunn

**Affiliations:** Psychology Department, University of British Columbia, Vancouver, British Columbia, Canada; University of Sydney, Australia

## Abstract

Evolutionary models of cooperation require proximate mechanisms that sustain prosociality despite inherent costs to individuals. The “warm glow” that often follows prosocial acts could provide one such mechanism; if so, these emotional benefits may be observable very early in development. Consistent with this hypothesis, the present study finds that before the age of two, toddlers exhibit greater happiness when giving treats to others than receiving treats themselves. Further, children are happier after engaging in *costly giving* – forfeiting their own resources – than when giving the same treat at no cost. By documenting the emotionally rewarding properties of costly prosocial behavior among toddlers, this research provides initial support for the claim that experiencing positive emotions when giving to others is a proximate mechanism for human cooperation.

## Introduction

Contrary to traditional economic theory that depicts human beings as fundamentally motivated by self-interest, people routinely engage in cooperative acts—from giving blood to donating to charity—that require them to incur personal costs for the benefit of others. Indeed, human survival and flourishing have depended on our species’ ability to work together to achieve feats that could not be achieved alone, such as hunting large prey and building shelter in inhospitable regions [1]. These behaviors present a puzzle that has intrigued social scientists for decades: whereas cooperation is beneficial once established at a population level [2]–[9], cooperation frequently requires that individuals engage in prosocial acts, facing a loss of resources and potential physical harm. What inspires and sustains such costly tendencies in individuals?

The prosocial behaviors that underlie cooperation could be supported entirely by cultural mechanisms, such as explicit teaching and the imitation of prominent role models. Although socialization almost certainly plays an important role in supporting human prosociality, this process may be complemented by an evolved proclivity for human beings to find prosocial behavior “self-rewarding.” Indeed, behaviors that are associated with positive emotions are more likely to be repeated [10], providing a powerful proximate mechanism that could sustain prosocial behavior even among individuals who are not fully socialized.

Supporting the possibility that humans may have evolved to find generous acts rewarding, adults from around the world report higher levels of happiness when spending money on others than when spending money on themselves [11], [12], and giving money to charity activates regions of the brain associated with processing reward [13]–[15]. Moreover, interventions encouraging participants to engage in acts of kindness have been shown to increase well-being among adults [16], [17]. Indeed, despite lay conceptions that children are inherently selfish [18]–[20], even young children engage in prosocial behavior: toddlers attempt to comfort individuals in distress [21], and assist others in achieving their goals, even at cost to themselves [22], [23]. Although these results are consistent with the hypothesis that giving to others is inherently rewarding for young children, no research has directly tested this central premise or explored the ontogenetic origins of the relationship between giving and happiness. Here, we investigate whether children under the age of two experience greater happiness when giving treats to others rather than receiving treats themselves. In addition, because forms of giving that require individuals to forfeit their own resources should be the most difficult to sustain, we examine whether children are happier when engaging in *costly giving* than when giving the same resource at no personal cost.

**Figure 1:**
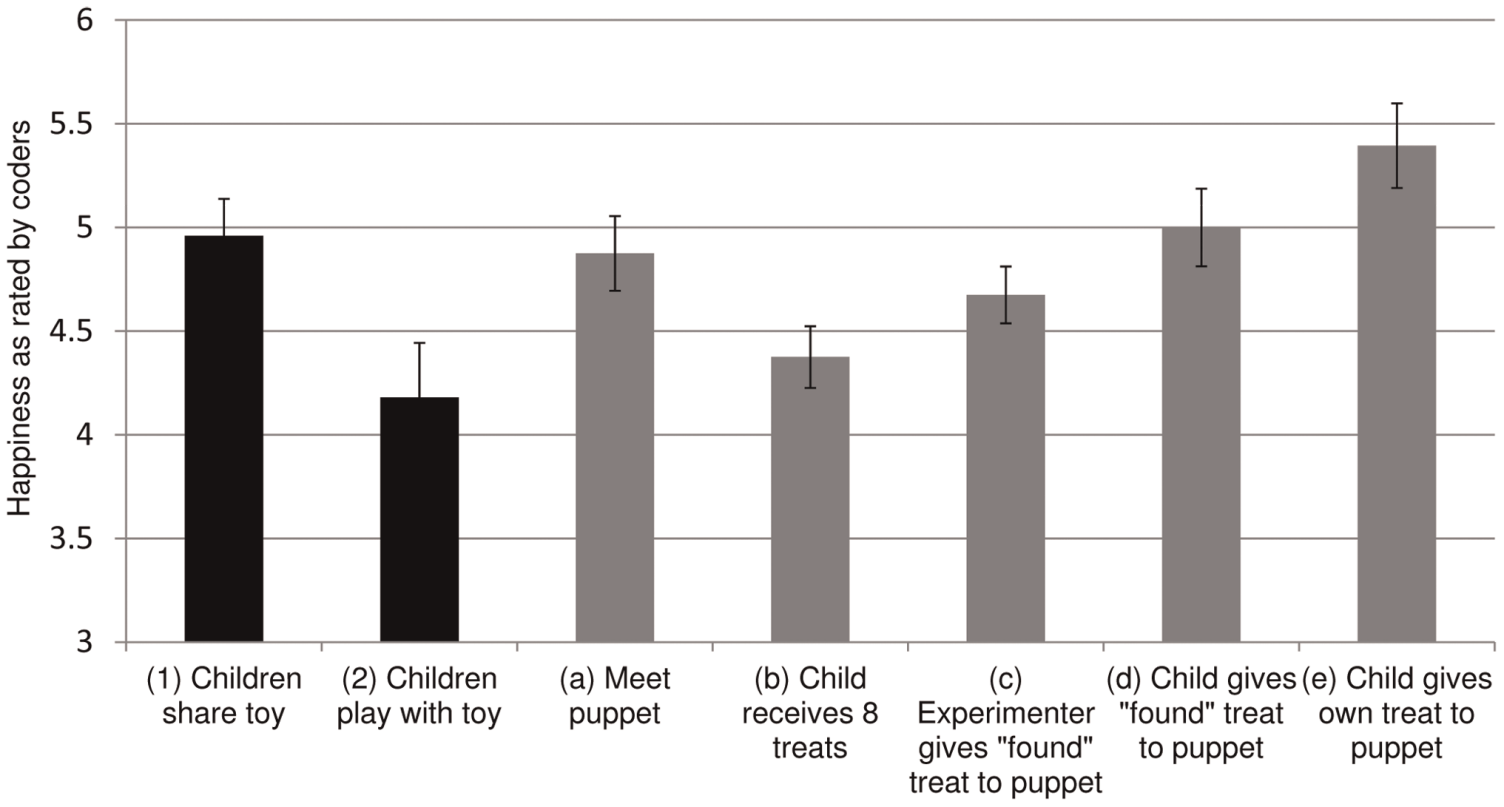
Happiness displayed in each preliminary study and main experiment condition. Happiness, as rated by coders, for children in the preliminary study and five phases of the main experiment. Error bars represent 95 percent confidence intervals around the mean.

As an initial test of the hypothesis that giving produces emotional benefits among young children, we examined past studies from our lab, in which toddlers played a social game that did or did not involve giving. Emotional expressions were videotaped and coded by two trained assistants (blind to hypotheses) for happiness on a seven-point scale (1-*not at all happy*; 7– *very happy*, α = .92); naive coder ratings have been shown to correlate highly (*r>*.95) with other validated measures of emotional coding, such as Baby FACS [24]. Specifically, twenty-three toddlers either (1) shared a toy with a puppet, to which the puppet responded positively, or (2) activated an appealing animal-sound toy that a puppet had taught them to use, to which the puppet responded positively; interactions with puppets are commonly used to provide a controlled but engaging situation for studying young children’s moral behavior [25], [26]. Consistent with the hypothesis that giving is emotionally rewarding in young children, toddlers who shared a toy with a puppet displayed greater happiness than toddlers who interacted with a toy and a puppet (Analysis of Variance (ANOVA) *F*(1,21)  = 6.13, *p<*0.03, *d = *1.02, Figure 1). Although these studies were not designed to compare giving and other positive social interactions, they provide suggestive evidence for the emotional benefits of giving in toddlerhood, and the impetus for a controlled experiment in which emotional expressions to giving and non-giving could be examined within individual toddlers.

In our main experiment, each toddler received treats and gave them away, under conditions in which giving was or was not personally costly. The experiment began with a warm-up phase designed to acclimate toddlers to the experimental situation. Each child was introduced to several puppets who “liked treats” and watched the experimenter give each one a treat (either Goldfish crackers or Teddy Grahams; one kind of treat was used in the warm-up and the other in the main study, with treat type counterbalanced). Puppets “ate” the treats placed in their bowls, by making “YUMMM” eating noises and pushing them through the bowls’ false bottoms. In addition, children gave treats to the puppets and received treats themselves (additional details in methods summary). We assumed that children would believe that the puppets ate and enjoyed the treats because previous research has shown that infants and toddlers attribute perceptual states, goals and desires to non-human agents [25], [27]–[30] (cf [31]). Indeed, research suggests that toddlers can distinguish between individual puppets’ preferences for different kinds of treats [32].

**Figure 2:**
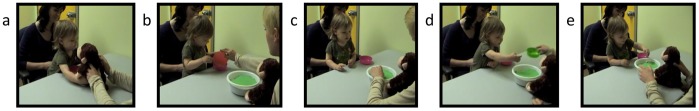
Five phases of the main experiment. Toddlers were (a) introduced to a puppet and (b) given eight treats. Then, in counterbalanced order, each toddler (c) watched as the experimenter gave one treat to the puppet, (d) was asked to give a “found” treat to the puppet, and (e) was asked to give one of their own treats to the puppet.

After the warm-up, participants moved to the testing phase. Children were (a) introduced to a new puppet (“Monkey”), encouraged to touch it, and told it liked treats. The experimenter said “Both you and Monkey have no treats right now,” to draw children’s attention to the limited nature of this resource. The experimenter then (b) “found” eight treats, said they were all for the child, and placed them all in the child’s bowl. Next, the experimenter (c) “found” a treat and gave it to the puppet, (d) “found” another treat and asked the child to give it to the puppet, and (e) asked the child to give the puppet a treat from the child’s own bowl (see Figure 2). Participants’ happiness during each phase was coded by the same research assistants using the same scale as in the preliminary study (average alpha  = .84). Phases (c) – (e) were counterbalanced; there were no significant order effects on children’s happiness (ANOVAs, *p*s >.095).

## Results

A repeated-measures ANOVA indicated that children’s happiness levels differed across the five study phases (*F*(4,72)  = 6.76, *p*<.001). Testing our key questions, we found that toddlers did not find giving aversive; rather, they exhibited greater happiness when giving treats to the puppet than when receiving treats themselves. This held true both in phase (d) when toddlers gave the puppet the treat that the experimenter “found” (repeated measures ANOVA *F*(1,18)  = 5.58, *p<*.04, *d = *0.88), and in phase (e) when they gave the puppet their own treat (repeated measures ANOVA *F*(1,18)  = 15.84, *p<*.005, *d = *1.35; Figure 1; sample video clip can be found online at http://cic.psych.ubc.ca/Example_Stimuli.html). Critically, a comparison of phases (d) and (e) revealed that toddlers were happier when giving away their own treat than when giving the “found” treat (repeated measures ANOVA, *F*(1,18)  = 4.52, *p*<.05, *d = *0.46). This difference emerged despite the fact that in phases (d) and (e) the child’s interaction with the puppet (i.e., taking a treat from a bowl and placing it in the puppet’s bowl) was exactly the same; the sole distinction between these phases was the source of the treat, with the child sacrificing his/her own treat only in phase (e). This comparison acknowledges the personal sacrifice often involved in prosocial behaviour, and suggests that rather than finding such acts aversive, children find them emotionally rewarding.

To test the possibility that children’s positive responses to giving were driven by the puppet’s reaction to receiving treats, our coders also rated how much enthusiasm the puppet displayed in each phase. Puppet’s enthusiasm was uncorrelated with children’s happiness in any phase (Pearson’s correlation coefficient *r*s from .00 to −.21, *p*s >.25), suggesting children’s happiness was not a function of puppet enthusiasm.

## Discussion

While previous research has demonstrated spontaneous helping among toddlers in the absence of explicit or implicit social demands [23], the present study provides the first evidence that giving to others makes young children happy-even happier than when they are receiving treats themselves. In addition, children’s differential happiness during costly and non-costly giving suggests that they (1) distinguished between the two, and (2) derived more happiness when giving involved sacrificing their own resources. This result is especially significant in that the ‘puzzle’ of prosocial behaviour toward non-kin requires explaining costly prosocial behaviour in particular; from our theoretical perspective, the emotional benefits of this form of giving may support such behavior despite its costs, whereas non-costly prosocial behaviour could be sustained in the absence of a powerful proximate mechanism.

While the role of socialization can almost never be completely ruled out, the present results support the argument that humans have evolved to find prosocial behavior rewarding. Although children may be socialized to engage in helping and sharing behavior before the age of two through praise and other forms of reinforcement, it seems unlikely that children’s nonverbal responses in this experiment solely reflected their anticipation of external rewards. Indeed, children’s happiness was unrelated to the puppet’s enthusiasm, and previous research has shown that external reinforcement of prosocial behavior actually *undermines* toddlers’ subsequent prosocial acts, both immediately [23] and two years later [33]. Finally, from a socialization perspective, it is difficult to explain our finding that the emotional benefits of giving are strongest when giving is costly; it seems unlikely that parents provide toddlers with differential reinforcement for costly versus non-costly forms of giving.

Future work examining whether giving leads to happiness in young children should replicate these findings with a larger sample. While the present research suggests the impact of giving on happiness is large (effect sizes between 0.46–1.35), replicating these findings with an additional sample would further support these claims. Future work should also examine whether giving to some targets produces greater emotional rewards than giving to others; for instance, perhaps toddlers would be happiest after giving to relatives or to people who had provided help in the past. Finally, given research suggesting that adults experience greater emotional benefits when they freely choose to help others than when they feel obligated to do so [34], research should also explore whether the emotional benefits of giving are greater when children give spontaneously, as opposed to giving in response to an experimenter’s suggestion.

We have argued that the warm glow of giving represents a proximate mechanism that encourages individuals to engage in prosocial behavior, even, or perhaps especially, when doing so is costly. In addition to simply reinforcing prosocial behavior, positive emotions facilitate constructive mindsets [35], [36], promote success in a wide range of domains [37], and have been linked to longevity [38], making happiness an especially adaptive proximate mechanism. For example, individuals who report experiencing more frequent positive emotion are more likely to seek out creative solutions to novel problems, excel professionally, get married, and live longer.

In sum, the present research speaks to the origins of human prosociality towards non-kin, a puzzle that has intrigued scientists for decades. By documenting the emotionally rewarding properties of costly prosocial behavior among children in the second year of life, this research provides foundational support for the claim that experiencing positive emotions when giving to others is a proximate mechanism for human cooperation and prosociality.

## Methods

All participants were healthy full-term toddlers recruited from local libraries, hospitals, and community events in Vancouver, Canada. Families were contacted and asked to bring their child into the lab when their children fell within the appropriate age window for a study. Public transportation or parking costs were paid for by the lab and children received either a t-shirt or small toy for their participation. All data collection was approved by the University of British Columbia’s Behavioral Research Ethics Board (H10–01808). Age and gender were unrelated to happiness ratings in all study phases (all *p*s >.18).

### Preliminary Study

Eleven toddlers (5 boys; *X*_age_  = 22 months, 1 day, range 21;16 to 22;20) participated in the giving interaction; 12 in the non-giving interaction (6 boys; *X*_age_  = 19 months, 27 days, range 19;13 to 20;8). Children were recruited for two separate studies investigating what infants offer to someone else (giving interaction) [39] and whether infants can distinguish between formerly helpful and unhelpful individuals when they need help (non-giving interaction) [40]. Giving and non-giving interactions represented the warm-up phase for each study, respectively. Children were allocated to the two studies based solely on their age. Children were later randomly assigned to different conditions within their experiment. Importantly, however, this condition assignment occurred *after* the giving and non-giving warm-up, meaning that participants were not treated differently based on condition during the time at which their responses were captured.

### Main Study

Twenty toddlers (11 boys, *X*_age_  = 22 months, 26 days, range 22;7 to 23;17) participated. Ten additional children were excluded for technical/experimental errors (5), fussiness (3), failure to complete the warm-up because of shyness (1), and refusal to share in the warm up (1). One child failed to complete phase (d), instead spontaneously giving his own treats, resulting in a missing data point.

The *warm-up* took approximately 3 minutes and acclimated children to the experimental situation. Three puppets were presented; children were told they could touch the puppets and that they liked treats. The puppets and the child each received a bowl, and a treat was placed in each one. Puppets “ate” their treat, by making “YUMMM!” noises and pushing it through a false bottom of the bowl. Children were allowed to eat their treat. The researcher then placed a bowl with four additional treats next to the child’s bowl. Children were asked to give a treat to each puppet. If children hesitated, the experimenter prompted the action by (a) pointing at the treat then the puppet’s bowl, (b) picking up the treat, (c) giving the treat to the child, (d) telling the child their parent/guardian approves, (e) asking the parent/guardian to hold the treat, (f) asking the parent/guardian to give the child the treat. Prompts were used only if needed; only one child required a prompt beyond b. As before, each puppet “ate” the treat after it was placed in their bowl. The final treat was given to the child. The *test period* took approximately 2 minutes and proceeded as already described.
